# PirB functions as an intrinsic suppressor in hippocampal neural stem cells

**DOI:** 10.18632/aging.203134

**Published:** 2021-06-13

**Authors:** Baiyang Liu, Wenjing Cheng, Dating Cheng, Jun Pu, Zhi Nie, Cuifeng Xia, Yongbin Chen, Cuiping Yang

**Affiliations:** 1 Key Laboratory of Animal Models and Human Disease Mechanisms of Chinese Academy of Sciences & Yunnan Province, Kunming Institute of Zoology, Kunming, Yunnan 650223, China; 2 Kunming College of Life Science, University of Chinese Academy of Sciences, Beijing 100049, China; 3 Kunming Medical University, Kunming, Yunnan 650500, China; 4 Center for Excellence in Animal Evolution and Genetics, Chinese Academy of Sciences, Kunming, Yunnan 650223, China

**Keywords:** paired immunoglobulin-like receptor B, PirB, Akt1, neural stem cells, NSCs, neurogenesis

## Abstract

Neural stem cells play pivotal roles during prenatal development and throughout life. Here, we report that Paired immunoglobulin-like receptor B (PirB) functions as a suppressor during brain neurogenesis in the adult mouse. PirB expression increased with age during development, and its deficiency promoted neural stem cell proliferation and differentiation *in vivo* and *in vitro*. Furthermore, we detected an increase in Type 1 neural stem cells in PirB-deficient mice compared to their wild-type littermates. PirB deficiency promoted stemness marker gene expression of Sox2 and KLF4 by activating Akt1 phosphorylation. These findings suggest that PirB inhibits the self-renewal and differentiation capacities of neural stem cells. Thus, PirB may have the potential to serve as a therapeutic target for treatment of reduced neurogenesis in adults due to aging or other pathological conditions.

## INTRODUCTION

Neural stem cells (NSCs) generate all major neural cell types in the central nervous system (CNS), including neurons, astrocytes, and oligodendrocytes [1–3]. NSCs persist in two locations in the adult brain to produce new neurons: the subventricular zone (SVZ) and the subventricular zone (SVZ). The SVZ generates neuroblasts that migrate over a great distance through the rostral migratory stream to the olfactory bulb and participate in normal olfaction functions, while SGZ NSCs in the hippocampal dentate gyrus migrate into the granular cell layer and produce neurons involved in modulating mood as well as short-term learning and memory [1]. NSCs are radial glia-like cells with an elaborate tree of processes in the granule cell layer and GFAP expression [4, 5]. However, an ongoing debate is whether neurogenesis occurs in areas of the adult mammal brain other than the SVZ and SGZ [6]. Adult neurogenesis is regulated by physiological and pathological activities at all levels, which includes adult NSCs proliferation, maturation, survival, differentiation, and integration of newborn neurons [2]. The stem cell pool is exhausted after development [7]. Extrinsic environmental signals and intrinsic signaling pathways regulate neurogenesis by activating quiescent NSCs, fate specification, new neuron development, and integration [8–12]. CNS resident cells, including peripheral immune cells, participate in functional regulation during hippocampal adult neurogenesis, and the inflammatory environment enhances NSC proliferation in the SGZ [13–15]. The phagocytosis-related protein Mfge8—secreted by activated microglia and astrocytes—also regulates the adult NSC pool [16, 17]. Interestingly, a role for the innate and adaptive immune systems in adult neurogenesis has been documented during aging [18]. However, whether immune signaling molecules regulate neurogenesis directly remains unknown.

Immune signaling is well known for its roles in pathogen response and tissue injury and is an emerging factor for controlling resident NSC behavior [19]. For example, the major histocompatibility complex class I (MHCI) proteins and MHCI receptors play important roles regulating neuronal plasticity [20]. Paired immunoglobulin-like receptors (PIRs), also known as leukocyte immunoglobulin-like receptors (LIR) or Ig-like transcripts (ILT) in humans, are expressed on B cells and myeloid lineage cells, and include the inhibitory PirB and activating isoform PirA, which bind to class I MHC molecules [21, 22]. PirB is crucial for inhibiting axon regeneration and synaptic plasticity, supporting its use as a target for axon regeneration therapies [23–28]. Interestingly, MHCI and PirB exacerbate brain injury following ischemia despite restricting synaptic plasticity in healthy neurons [29]. MHCI and PirB are upregulated in the neuroglial of the hippocampus in aged rats [30]. Kim et al. reported that PirB and its human ortholog LilrB2 (leukocyte immunoglobulin-like receptor B2) are receptors for soluble β-amyloid (Aβ) oligomers—key mediators for cognitive malfunction in Alzheimer disease (AD) [31]. Srinivas Ramasamy et al. reported that blocking PirB by a soluble PirB ectodomain reduced neurosphere formation [32]. Thus, although PirB is involved in neural development, whether intrinsic PirB can regulate the stemness maintenance of NSCs is unknown.

The phosphatidylinositide 3 kinases (PI3Ks)/Akt serine/threonine kinase (AKT) signaling cascade regulates cell proliferation, survival, and metabolism processes. Recent findings suggest PI3K/AKT signaling control pluripotency and differentiation by preserving the self-renewal and differentiation ability of pluripotent stem cells in a Sox2 activation-dependent manner [33–37]. Importantly, multiple studies demonstrated that PirB functions as a potential suppressor of PI3K/Akt signaling pathway [26, 38]. In this study, we assessed whether intrinsic PirB residing in the hippocampal progenitor niche is important for postnatal dentate gyrus development and sought to decipher its underlying mechanism. Our findings support the use of PirB as a novel therapeutic target to reactivate decreased adult neurogenesis due to aging or pathological conditions.

## RESULTS

### PirB increases with age in hippocampal NSCs

**Figure 1:**
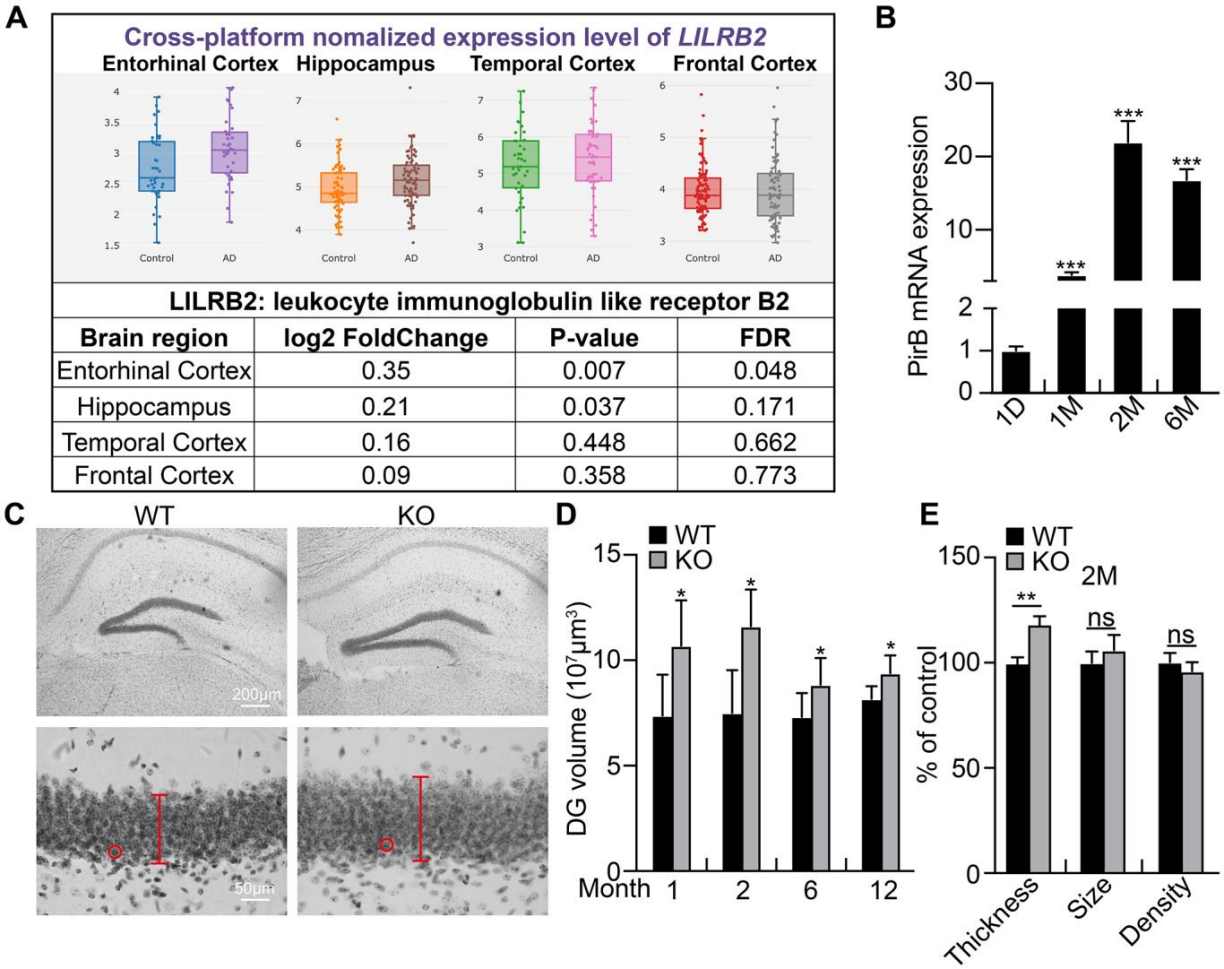
**PirB increases with age in hippocampal neural stem cells.** (**A**) Expression pattern of PirB human ortholog LilrB2 in the central nervous system as analyzed by a web source dataset: http://www.alzdata.org/Normalized_differential.php. (**B**) qRT-PCR of PirB mRNA expression in NSCs isolated from mice of different ages. (**C**–**E**) Dentate gyrus (DG) volume quantified over time using three-dimensional volumetric reconstructions following Nissl staining and shown to be enlarged in PirB-depletion mice (PirB KO) compared to wild type (WT) at different ages. *n* ≥ 3. Red lines and circles: thickness of the granular layer and the size of single cells, respectively. (**E**) Neuronal cell number increased upon PirB depletion based on the thicker granular layer and unaffected cell density or size. Data is shown the percentage of control. Means ± SEM, ^*^P < 0.05; ^**^P < 0.01; ^***^P < 0.001; ns = no significant difference by the *t*-test.

PirB is distributed in various hematopoietic cells including B cells, mast cells, macrophages, neutrophils, and dendritic cells [39, 40]. PirB is also found in different regions of the injured central nervous system, including the cerebral cortex, hippocampus, cerebellum, olfactory ensheathing neurons, axon cells, neuropil, spinal cord, and rubrospinal neurons [23, 24]. To determine whether PirB is expressed in NSCs and regulates neurogenesis, we focused on the hippocampal region. We investigated PirB regulation of hippocampus development by comparing the dentate gyrus volume between littermate wild-type and PirB knockout mice. Consistent with previous findings [30], we observed that PirB mRNA expression increased in brain NSCs as they aged (Figure 1A–1B). Furthermore, the dentate gyrus volume was dramatically enlarged in PirB knockout mice versus wild-type mice and its granular layer contained more granule neurons (Figure 1C–1D). However, neither the total cell density nor size was affected upon PirB depletion (Figure 1E). These results suggest that PirB is critical for hippocampal neurogenesis *in vivo*.

### PirB depletion promotes NSC self-renewal and differentiation

**Figure 2:**
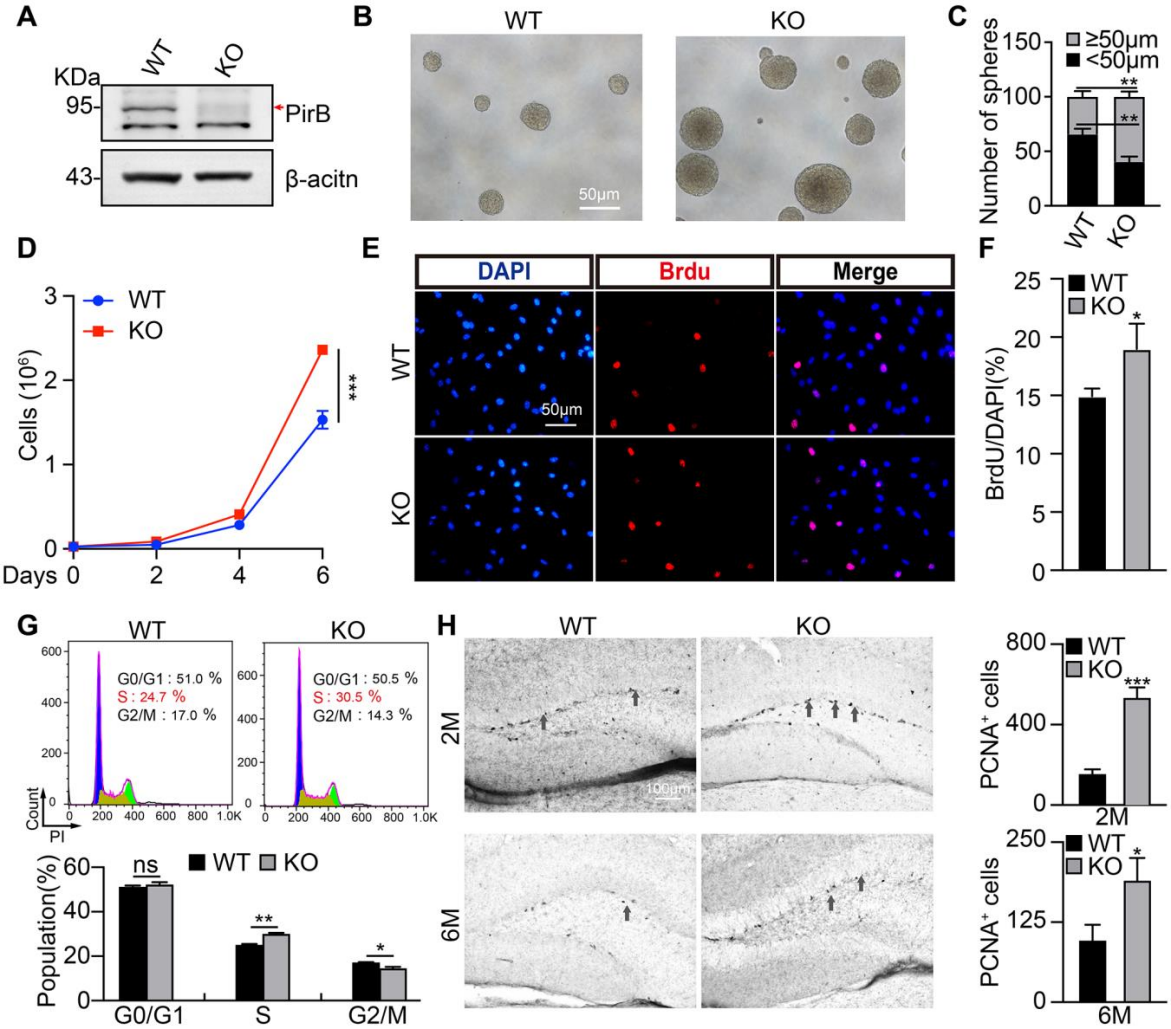
**PirB knockout promotes NSC stemness maintenance.** (**A**) PirB knockout (KO) was verified by western blot. Arrow: PirB molecular weight. (**B**–**C**) Neurospheres from wild-type and PirB-depleted animals indicate that PirB-deficient progenitors show an increased self-renewal capacity. (**D**) PirB depletion promoted cell proliferation. (**E**–**F**) PirB deficiency increased DNA synthesis in NSCs as shown by BrdU incorporation. Scale bar: 50 μm. (**G**) PirB knockout promoted cell cycle transition in NSCs, as measured by propidium iodide staining and flow cytometry. (**H**) 3,3’-diaminobenzidine (DAB) staining for PCNA shows the hippocampal dentate gyrus cell proliferation in 2- and 6-month-old mice, respectively. *n* ≥ 3. Means ± SEM, ^*^P < 0.05; ^**^P < 0.01; ^***^P < 0.001; ns = no significant difference by the *t*-test.

**Figure 3:**
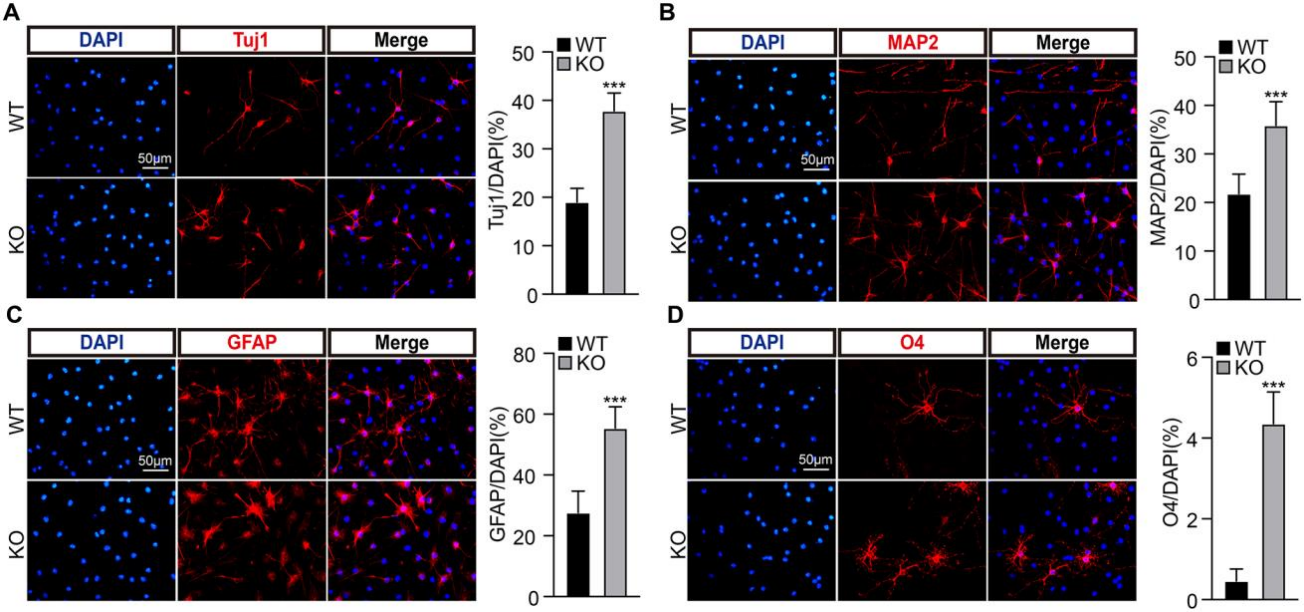
**PirB knockout promotes NSC differentiation ability.** (**A**–**B**) PirB knockout promotes the differentiation of NSCs into neurons. Tuj1 and MAP2 marks immature postmitotic neurons and mature neurons, respectively. PirB knockout promotes the differentiation of NSCs into (**C**) astrocytes and (**D**) oligodendrocytes. Means ± SEM, ^*^P < 0.05; ^**^P < 0.01; ^***^P < 0.001 by the *t*-test.

To examine whether PirB regulates neurogenesis via modulating neural stem cell behavior, we decided tested the self-renewal of NSCs. We first cultured NSCs isolated from postnatal day 7 hippocampal dentate gyrus in wild-type or PirB knockout mice, respectively, and examined their PirB protein expression by western blot (Figure 2A). Next, we obtained neurosphere cells using culture conditions favoring stem cell growth. We discovered that the number and sizes of neurospheres (diameter ≥ 50 μm) derived from PirB knockout-NSCs increased in comparison to wild-type neurospheres (Figure 2B–2C). To validate whether the cell cycle transition of NSCs was regulated by PirB, we performed cell proliferation and BrdU incorporation assays. An increment of cell numbers and BrdU-positive cells in PirB knockout was detected (18% vs. 14%) compared to wild-type NSCs (Figure 2D–2F). Flow cytometric analysis showed that PirB deficiency led to an increased S-phase cell population (Figure 2G). To corroborate these findings *in vivo*, we performed IHC staining using the Proliferating Cell Nuclear Antigen (PCNA) as a proliferation marker [41, 42]. We found an increase in PCNA-positive cells in the PirB-depleted hippocampal regions of 2- and 6-month-old mice compared to the wild-type controls (Figure 2H). In addition, we found an increase in cells expressing Tuj1, MAP2, GFAP, and O4+ markers for immature postmitotic neurons, mature neurons, astrocytes, and oligodendrocytes, respectively, after PirB depletion (Figure 3A–3D). These results suggest that PirB knockout promotes the self-renewal and differentiation abilities of NSCs.

### PirB knockout promotes NSC stemness maintenance *in vivo*

**Figure 4:**
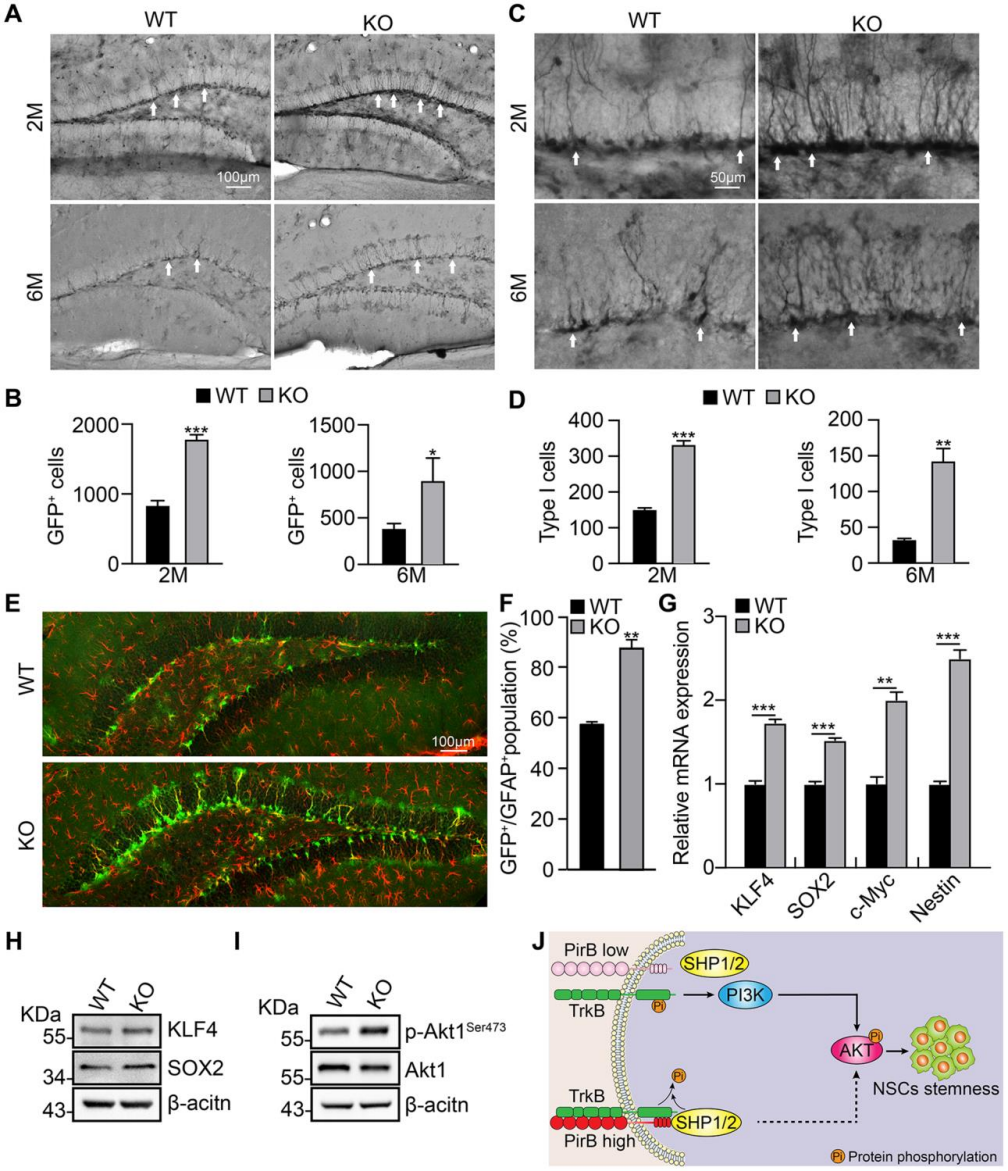
**PirB depletion increases the NSC pool *in vivo* through Akt1 signaling.** (**A**–**B**) Neural stem/progenitor cell (GFP-expressing cell) number quantified in the dentate gyrus of mice over time. *n* ≥ 3. (**C**–**D**) Type 1 cells (arrows) were increased in PirB-depleted mice compared with the wild-type control group; *n* ≥ 3. (**E**–**F**) PirB knockout increased Type 1 early progenitors in 2-month-old mice. (Green: GFP-positive; red: GFAP positive); *n* ≥ 3. (**G**) qRT-PCR relative mRNA expression of KLF4, SOX2, c-MYC, and Nestin. (**H**) Increases in stemness marker genes KLF4 and Sox2 were verified by western blot. (**I**) Akt1 phosphorylation was increased upon PirB depletion as shown by western blot. (**J**) Working model for PirB in NSCs. PirB deficiency promotes Akt1 phosphorylation through reducing recruitment and binding of Src homology 2-containing protein tyrosine phosphatase (SHP)-1 and SHP-2 to inactivate TrkB. This results in constitutive activation of the Akt1 signaling pathway and increased NSC self-renewal. Means ± SEM, ^*^P < 0.05; ^**^P < 0.01; ^***^P < 0.001 by the *t*-test.

To validate the functional role of PirB *in vivo*, we crossed PirB knockout mice with Nestin-GFP mice, in which the GFP-positive cell population indicates the NSCs population [7]. More GFP-positive cells were detected in the PirB-knockout hippocampal dentate gyrus of 2- and 6-month-old mice than wild-type mice (Figure 4A–4B). Morphological analysis showed that PirB deletion resulted in more glial-like GFP-positive cells (Figure 4C–4D), which indicates more type I NSCs in the PirB knockout mice than wild-type mice. Furthermore, representative immunostaining results showed that the Nestin-GFP+/GFAP+ double positive cell population (type I NSCs, [7]), was dramatically increased in PirB-deficient mice compared to wild-type mice (Figure 4E–4F).

### PirB inhibits Akt1 phosphorylation in NSCs

We performed real-time qRT-PCR and western blot assays to examine NSC expression of stemness marker genes KLF4, Sox2, c-Myc, and Nestin. We found that these genes were upregulated in NSCs upon PirB depletion versus the wild-type control (Figure 4G–4H). We also discovered that PirB knockout promotes Akt1 phosphorylation (Figure 4I), which is consistent with previous reports of PirB suppressing the PI3K/Akt signaling pathway [26, 38]. These findings suggest that PirB suppresses the self-renewal ability of NSCs via abrogating the PI3K/Akt signaling pathway.

## DISCUSSION

PirB was first identified in the immune system and shown to negatively regulate the peritoneal B1 cell compartment, humoral responses to TI antigens, and T_H_2 responses to TD antigens [40]. It was then identified as one of the age-related hippocampal changed genes, whose expression was induced in the CA1 and dentate gyrus of aged rats [30]. PirB can also act as receptor for the oligomeric forms of Aβ, the loss of which reduces damage caused by Aβ accumulation in the hippocampus of mice [31].

In this study, we found that PirB is expressed in hippocampal adult NSCs and intrinsic PirB depletion leads to increased NSC self-renewal and proliferation *in vitro*. Additionally, the PirB depletion enlarged the hippocampus and increased type I NSCs *in vivo*. We demonstrated that PirB knockout promotes the gene expression of stemness markers. Previous studies showed that a balanced expression of TrkB and PirB is critical for axon growth after injury, while TrkB is important for PI3K/Akt signaling activation [25]. In line with these findings [25], we observed that PirB deficiency promotes Akt1 phosphorylation, possibly through reducing recruitment and binding of Src homology 2-containing protein tyrosine phosphatase (SHP)-1 and SHP-2 to inactivate TrkB (Figure 4J).

Multiple studies indicate the suppressive role of PirB in the immune and central nervous systems after injury or advanced aging. However, the physiological roles of PirB during normal development should be further characterized. In addition, the reason for PirB induction in hippocampal adult NSCs with advanced aging remains unknown. To decipher the intrinsic and extrinsic mechanistic roles of PirB, the upstream regulators controlling PirB expression need to be uncovered, and the functional role of PirB during embryonic development should also be clarified. In summary, our results suggest that selectively blocking PirB might be a promising therapeutic strategy for elderly or other neurodegenerative patients in the future.

## MATERIALS AND METHODS

### Animals

All mice were humanely housed and cared for in the Animal Resource Center at the Kunming Institute of Zoology. The mice used for this work were wild-type and/or PirB-deficient mice crossed with Nestin-GFP mice on a C57/Bl6 background [7].

### Cell culture

The NSCs were prepared as previously described [7]. Briefly, wild-type or PirB-deficient dentate gyrus were isolated and digested with activated papain solution, and then grown in serum-free medium containing 20 ng/ml of epidermal growth factor, 20 ng/ml of fibroblast growth factor, 1X N2 supplement (Gibco), 1X B27 supplement (Gibco), 10 μg/ml of heparin, and 1% penicillin/streptavidin. For the cell proliferation assay, 5 × 10^4^ neural progenitor cells per well were plated on 8-well chamber slides (Thermo) and treated with 10 μM of 5-bromo-2′-deoxyuridine (BrdU) for 15 minutes to label dividing cells in growth medium. The BrdU-positive cells were counted with Image J software. For the differentiation assay, 5 × 10^4^ neural progenitor cells per well were plated onto 8-well chamber slides and cultured for three days in differentiation medium: serum-free medium without epidermal growth factor and fibroblast growth factor. The Tuj1+, MAP2+, GFAP+ and O4+ cells were counted with Image J software.

### RNA isolation and qRT-PCR

Total RNA isolated from mice brains were transcribed to cDNA using the SuperScript First-Strand Synthesis system (Takara) for RT-PCR analysis by Faststart Universal SYBR Green Master (Roche) with an ABI 7500 Real-time PCR system (Applied Biosystems). The relative gene amounts were normalized to actin. Primer sequences for real time PCR were: PIRB-F: 5′-CAATCAGGCTGCCGAATCT-3′, PIRB-R: 5′-CCGCCAGAGTAGCATATACAC-3′; ACTIN-F: 5′-GCGGACTGTTACTGAGCTGCGT-3′, ACTIN-R: 5′-TGCTGTCGCCTTCACCGTTCC-3′; SOX2-F: 5′-CACAGATGCAACCGATGCA-3′, SOX2-R: 5′-GGTGCCCTGCTGCGAGTA-3′; KLF4-F: 5′-CACACAGGCGAGAAACCTTACC-3′, KLF4-R: 5′-CGGAGCGGGCGAATTT-3′; NESTIN-F: 5′-CCAGAGCTGGACTGGAACTC-3′, NESTIN-R: 5′-ACCTGCCTCTTTTGGTTCCT-3′; C-MYC-F: 5′-AATCCTGTACCTCGTCCGAT-3′, C-MYC-R: 5′-TCTTCTCCACAGACACCACA-3′.

### Immunohistochemistry and immunocytochemistry

All animals were deeply anesthetized, perfused, and stained as previously described [7]. After perfusion, the brain was isolated, post-fixed overnight with 4% PFA, and sectioned with a vibratome at 50 μm intervals by embedding in 3% agarose in PBS (Leica, VT1000S). All sections through the hippocampus were collected in PBS containing 0.1% NaN_3_ and stored at 4°C. Every twelfth section of brain tissue was permeabilized with 0.3% Triton X-100 in PBS, blocked for at least 2 hours at room temperature with 10% normal goat serum, and incubated with primary antibodies followed by secondary antibodies. For immunocytochemistry analysis, cells were fixed with 4% PFA for 20 minutes at room temperature, blocked with 10% normal goat serum in PBS containing 0.1% Tween20 for 1 hour, and incubated with primary then secondary antibodies. The primary antibodies used were: rabbit anti-GFAP (DAKO, 1:2000), rabbit anti-GFP (Proteintech, 1:500), rat anti-BrdU (Abcam, 1:500), mouse anti-PCNA (Santa Cruz, 1:200), mouse anti-Tuj1 (Sigma, 1:1000), rabbit anti-MAP2 (Millipore, 1:1000), rabbit anti-GFAP (DAKO, 1:1000), and mouse anti-O4 (R&D System, 1:1000). Fluorescent-conjugated secondary antibodies were used (Invitrogen, 1:1000). Biotinylated-conjugated anti-species IgG (Vector Laboratories, 1:500) were used for peroxidase/diaminobenzidine (DAB) staining in stereology analysis.

### Stereological quantification

Cell counts were performed using an unbiased stereological analysis (Stereo-Investigator version 8, MBF Bioscience). For the analysis, every twelfth section of brain tissue was quantified. An unbiased counting frame was utilized with the Stereo Investigator software. The contours were traced in low magnification (10×), counting frames and the guard zones of all sections were set at 100 μm × 100 μm and 30-μm top and bottom, respectively. Cells were counted at high magnification (40×). DAB sections stained for GFP were also used to quantify the number of highly arborized dendritic tree morphology Type I cells for wild-type and PirB deficient mice [7]. For dentate gyrus volume quantification, the sections were stained with Nissl solution and estimated by the Cavalieri Estimator.

### Cell cycle analysis

Neural stem and progenitor cells were cultured as a monolayer and isolated into single cells before fixation in 70% ice-cold ethanol. Cell pellets were resuspended in 500 μl of staining buffer containing propidium iodide after a PBS rinse and analyzed by flow cytometry (BD Pharmingen).

### Statistical analysis

All statistical analysis was done in GraphPad Prism (Version 7.01; GraphPad Software Inc., La Jolla, CA, USA). *T*-tests (for paired data) or one-way ANOVA (for non-parametric) analysis were used for data analysis. All data shown represent the mean ± S.E.M. and a *P* value <0.05 indicates statistical significance.
